# Medial Patellofemoral Ligament Reconstruction Using Double Limbed Rectus Femoris Autograft

**DOI:** 10.1002/atn2.70009

**Published:** 2026-05-17

**Authors:** Vinicios Barreto Melo, Saulo Santos Blunk, Sérgio Marinho de Gusmão Canuto, Lorran Coque Fonseca, Camilo Partezani Helito, Victor Bezerra de Menezes Monnerat, Pedro Baches Jorge

**Affiliations:** ^1^ Knee Institute of Cachoeiro de Itapemirim Cachoeiro de Itapemirim Brazil; ^2^ Ortoclinica Maceió Brazil; ^3^ Saint Francis of Assis Children's Hospital Cachoeiro de Itapemirim Brazil; ^4^ Institute of Orthopedics and Traumatology Hospital das Clínicas Faculty of Medicine University of São Paulo (IOT–HCFMUSP) São Paulo Brazil; ^5^ Dona Lindu State Hospital of Traumatology and Orthopedics Paraiba do Sul Brazil; ^6^ Sports Trauma Group Department of Orthopedics and Traumatology Irmandade da Santa Casa de Misericórdia de São Paulo São Paulo Brazil

## Abstract

Medial patellofemoral ligament reconstruction is routinely performed when conservative treatment fails and patients present with recurrent patellar dislocations. Most described techniques require fixation or some type of bony procedure on the patella. The technique using a rectus femoris tendon graft, harvested with an open stripper and left attached to the patella, provides better anatomical correspondence with the native medial patellofemoral ligament and adequate graft thickness. Furthermore, it can be performed in skeletally immature patients without the need for fixation materials or bone tunnel creation.

## Graphical abstract

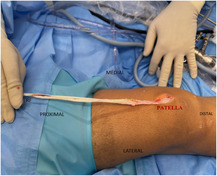

**VIDEO 1:** Patient is in the supine position, right knee, and the arthroscopic portal was anteromedial. Medial patellofemoral ligament reconstruction using rectus femoris graft: surgical technique description. We begin the physical examination by showing patellar lateralization in knee extension. During flexion, a significant patellar instability is observed. The surgical procedure starts with a 3 cm superomedial parapatellar incision, followed by layer‐by‐layer dissection and exposure of the rectus femoris tendon. After isolating the rectus femoris, the medial strip of the tendon is harvested with a width of 5 mm, preserving its patellar insertion. The tendon is then removed using an open stripper, with a graft length of approximately thirty centimeters. A subperiosteal dissection is carried proximally along the tendon to the superior third of the patella. The proximal portion of the rectus femoris (RF) tendon is then curved over the patella and passes under the vastus medial oblique muscle. With the graft folded over, the diameter using double limber RF autograft is measured for femoral tunnel preparation. A tenodesis is performed between the proximal portion of the graft and its distal portion in the medial region of the patella, ensuring adequate patellar fixation and mobility with the new ligament. A medial approach is made for femoral tunnel preparation. With the knee flexed and fluoroscopic imaging in the lateral view, a guidewire is advanced through Schöttle's point. After radiographic confirmation of the femoral entry site, the femoral tunnel is drilled. The prepared graft is then tunneled through the second layer of the knee (between the joint capsule and the medial retinaculum) toward the femur. With the knee flexed at approximately 30°, the graft is passed into the femoral tunnel, and its free end is secured with a bioabsorbable interference screw (Arthrex, Naples, FL, USA). The quadriceps tendon, vastus medialis obliquus, and the reconstructed medial patellofemoral ligament are then sutured. After reconstruction, the patella shows stability throughout the entire range of knee flexion. Video content can be viewed at https://doi.org/10.1002/atn2.70009.

Patellofemoral instability is a painful and often chronic condition following acute patellar dislocation.^1^, ^2^, ^3^, ^4^, ^5^ Its incidence ranges from 29 to 43 per 100,000 people per year during childhood and adolescence, and up to 10 times more frequent than in adults.^5^, ^6^ Traumatic patellar dislocation typically occurs in young, active patients, with recurrence rates reported as high as 50%.^4^, ^5^, ^6^, ^7^

Several anatomical factors contribute to patellar instability, including trauma, generalized ligamentous laxity, genu valgum, increased femoral anteversion, excessive tibial torsion, trochlear dysplasia, patella alta, and medial patellofemoral ligament (MPFL) insufficiency or rupture.^3^ The MPFL is recognized as the primary medial stabilizer of the patella,^1^, ^3^, ^4^, ^5^, ^6^, ^8^, ^9^ and studies show it is torn in 95% to 100% of patients after a first acute patellar dislocation.^3^, ^4^, ^6^, ^7^, ^9^, ^10^, ^11^

Anatomic MPFL reconstruction is essential to restore normal knee kinematics. MPFL reconstruction is one of the most frequently used procedures for recurrent patellar instability and may be combined with other alignment procedures when necessary.^4^, ^9^, ^12^ The MPFL plays a key role in guiding the patella medially during the first 20° to 30° of knee flexion, as shown in multiple clinical and biomechanical studies.^4^, ^7^, ^9^, ^12^

MPFL reconstruction was first described by Ellera Gomes in 1992 as an alternative treatment for chronic lateral patellar instability.^13^ Since then, several techniques have been proposed, using different grafts such as the gracilis tendon,^1^, ^14^, ^15^ quadriceps tendon,^10^, ^16^, ^17^, ^18^, ^19^, ^20^ patellar tendon,^21^ and even fascia lata. The macroscopic appearance of a strip of quadriceps tendon rectus femoris (RF) more closely resembles the native MPFL than hamstring tendon grafts.^6^, ^10^, ^21^

Despite technical variations, many surgeons use hamstring tendons for MPFL reconstruction, attaching them to the patella with anchors or bone tunnels^4^, ^6^ (Table 1). However, complications such as patellar fracture, implant loosening, and failure due to improper graft tensioning are relatively familiar with this approach.^4^, ^6^, ^22^ Although hamstring tendons have adequate mechanical properties, they do not ideally replicate the anatomical characteristics of the native MPFL.^4^

**TABLE 1:** Advantages and Disadvantages of MPFL Reconstruction With RF Graft

**Advantages**	**Disadvantages**
• Anatomical similarity between the MPFL and the rectus femoris tendon. • No need for fixation materials in the patella. • No patellar tunnel creation. • Reduced risk of patellar complications. • More anatomic and potentially isometric reconstruction. • Suitable for MPFL revision surgeries. • It can be performed in skeletally immature patients.	• Requires fluoroscopy. • Technical expertise is needed for rectus femoris harvesting.

MPFL, medial patellofemoral ligament; RF, rectus femoris.

The use of the quadriceps tendon as a graft has gained popularity. Several authors have reported encouraging clinical results with MPFL reconstruction using autologous quadriceps tendon.^10^, ^16^, ^17^, ^18^, ^19^ For quadriceps graft harvesting, some surgeons use only the superficial tendon layer,^7^, ^16^, ^18^, ^21^ whereas others prefer a full‐thickness central strip up to 10 cm in length.^7^, ^16^, ^18^, ^21^ When a superficial pedicled and inverted strip of quadriceps tendon is used (maintaining patellar insertion), patellar drilling or bony fixation is unnecessary, reducing the risk of postoperative iatrogenic patellar fracture.^4^, ^22^

## SURGICAL TECHNIQUE

The patient is placed supine under general anesthesia with peripheral nerve block (adductor canal). A standard diagnostic arthroscopy is performed to assess intra‐articular structures and evaluate patellar and trochlear cartilage (anterolateral arthroscopic portal). A longitudinal incision of approximately 3 cm is made in the suprapatellar region of the affected knee. After careful dissection of the subcutaneous and fascial planes, the proximal portion of the  RF tendon is identified.

**FIGURE 1:**
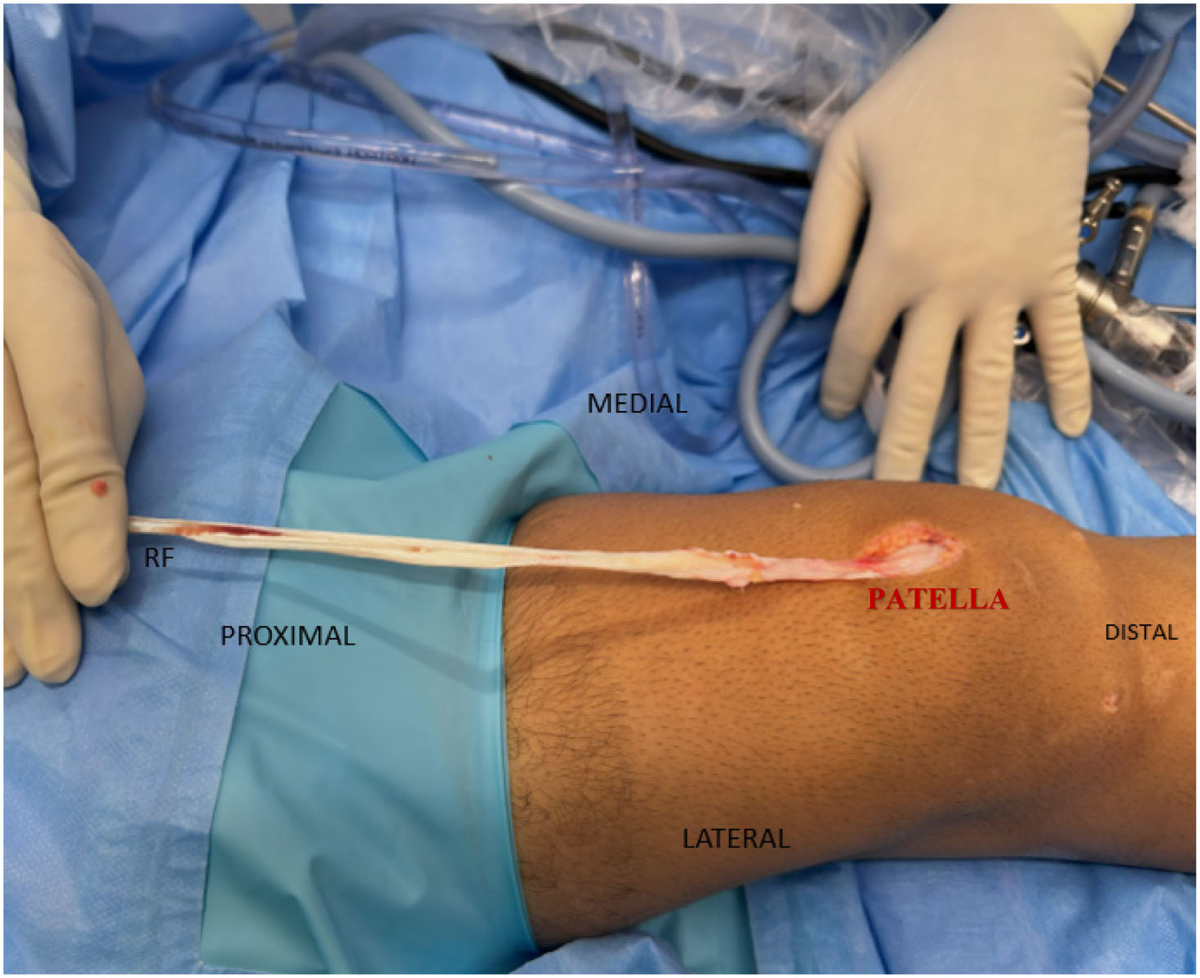
Patient is in the supine position, right knee, and the arthroscopic portal was anteromedial. Rectus femoris tendon removed with open stripper, keeping its distal insertion fixed to the patella.

**FIGURE 2:**
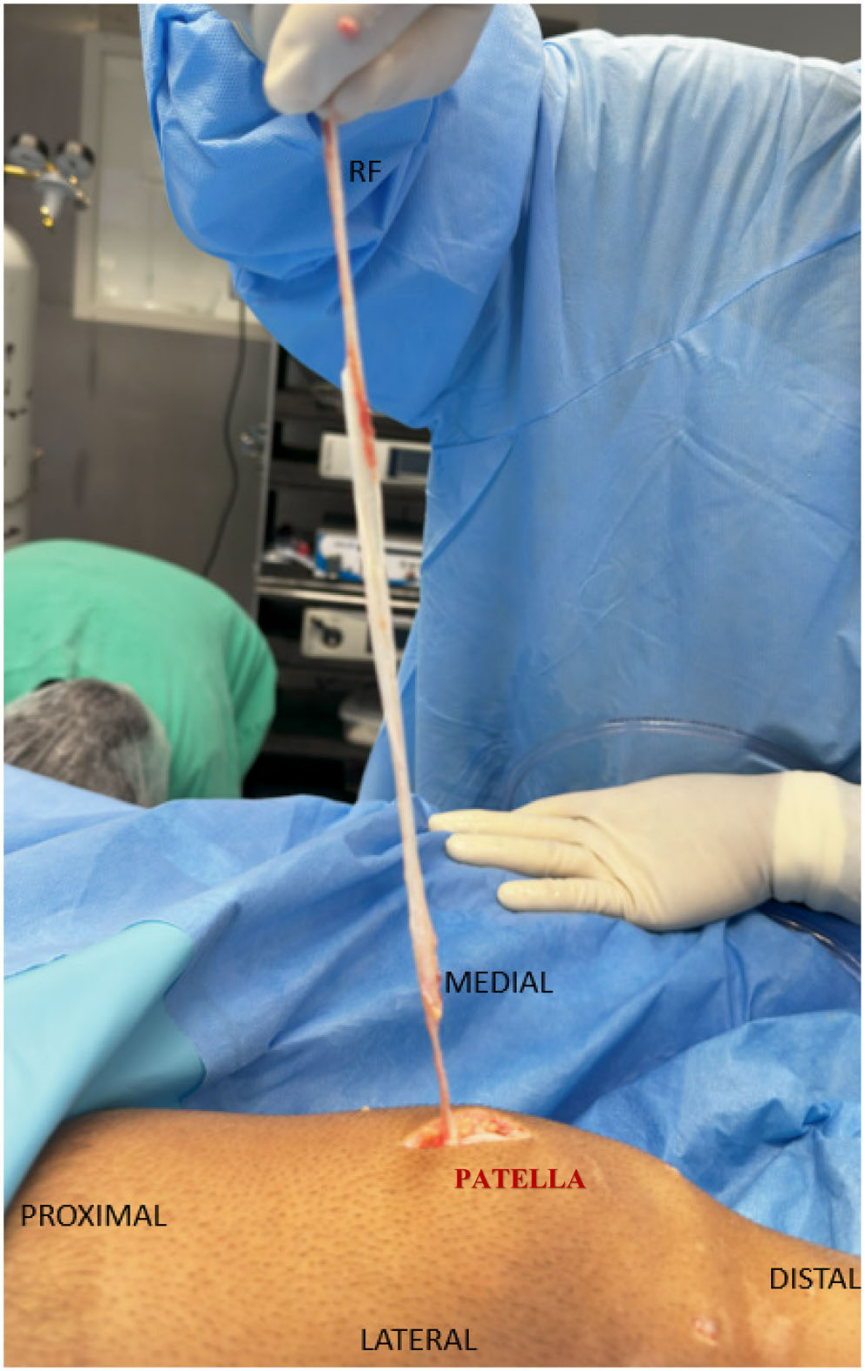
Patiente in supine position, left knee, showing rectus femoris graft removed with approximately 30 cm in length fixed to the patella for medial patellofemoral ligament reconstruction.

A medial strip of the RF tendon, approximately 5 to 7 mm wide, is fashioned with a scalpel. After isolating the RF, proximal harvesting is performed using an open stripper, preserving distal insertion at the patella (Figure 1, Video 1). Subperiosteal dissection is carried out along the femur to approximately the upper third of the patella, releasing the graft from adjacent deep tissues to obtain a length of about 30 cm (Figure 2, Video 1).

**FIGURE 3:**
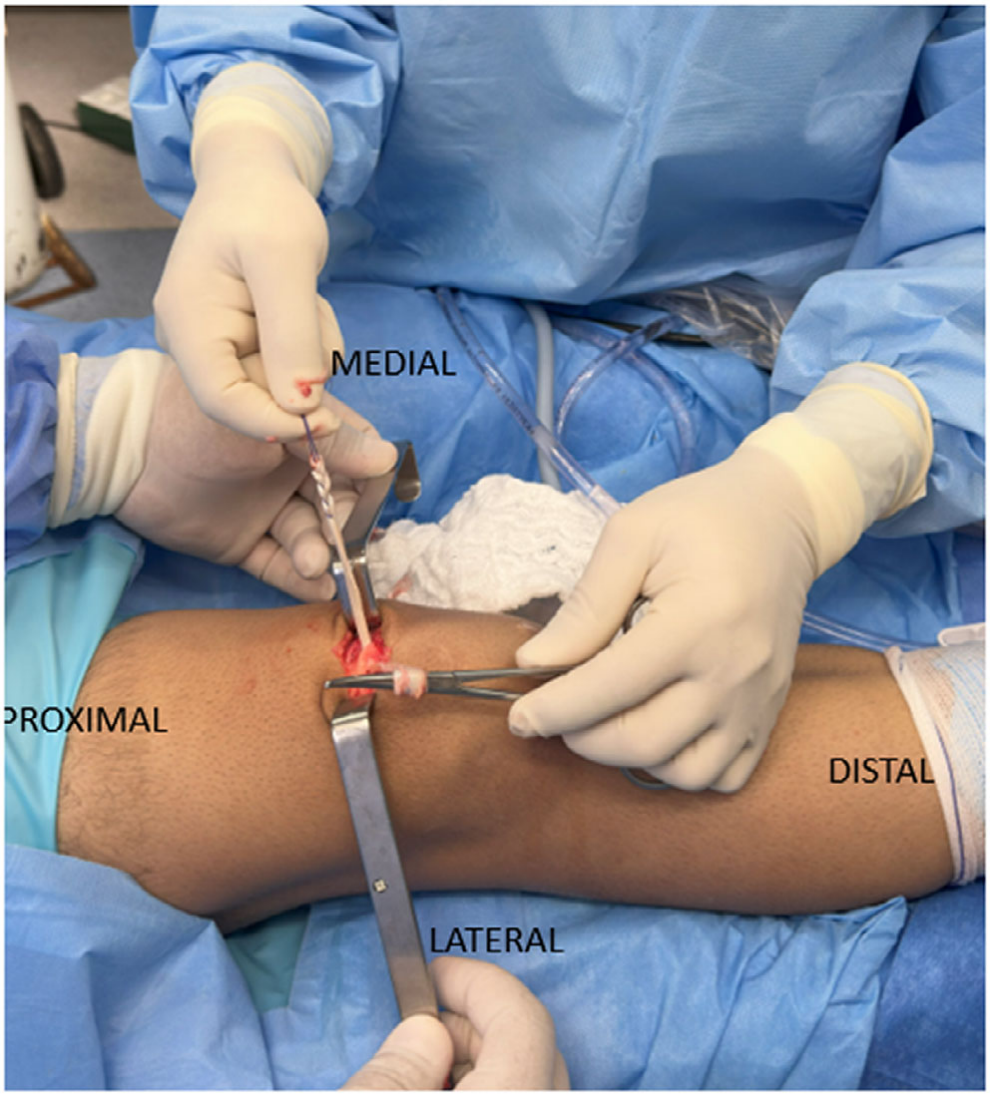
Patiente in supine position, left knee, showing the proximal free end of the RF tendon is then curved over the patella and passed beneath the vastus medialis obliquus muscle.

**FIGURE 4:**
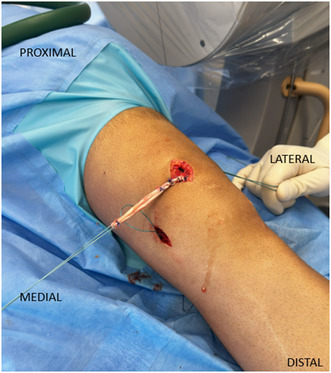
Patiente in supine position, left knee, showing the graft is folded onto itself (DOUBLE LIMBED), and tenodesis is performed by suturing the distal end to the anteromedial patellar tendon region, reinforcing patellar insertion.

The proximal free end of the RF tendon is then curved over the patella and passed beneath the vastus medialis obliquus muscle, creating an anatomic path toward the medial femur (Figure 3, Video 1). The graft is folded onto itself, and tenodesis is performed by suturing the distal end to the anteromedial patellar tendon region, reinforcing patellar insertion (Figure 4, Video 1).

**FIGURE 5:**
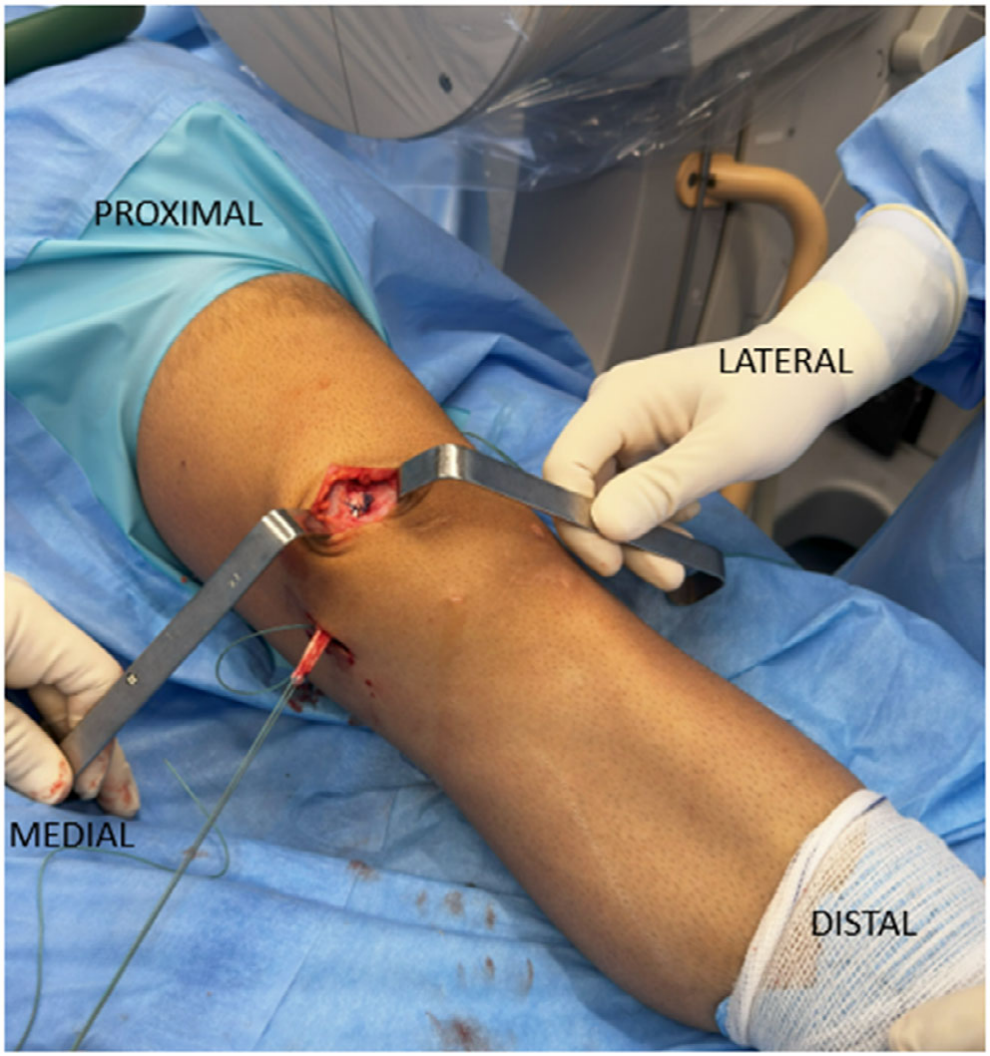
Patiente in supine psition, left knee, showing the prepared double rectus femoris graft for reconstruction of the medial patellofemoral ligament is tunneled through the second layer of the knee (between the joint capsule and the medial retinaculum) towards the medial epicondyle of the femur.

**FIGURE 6:**
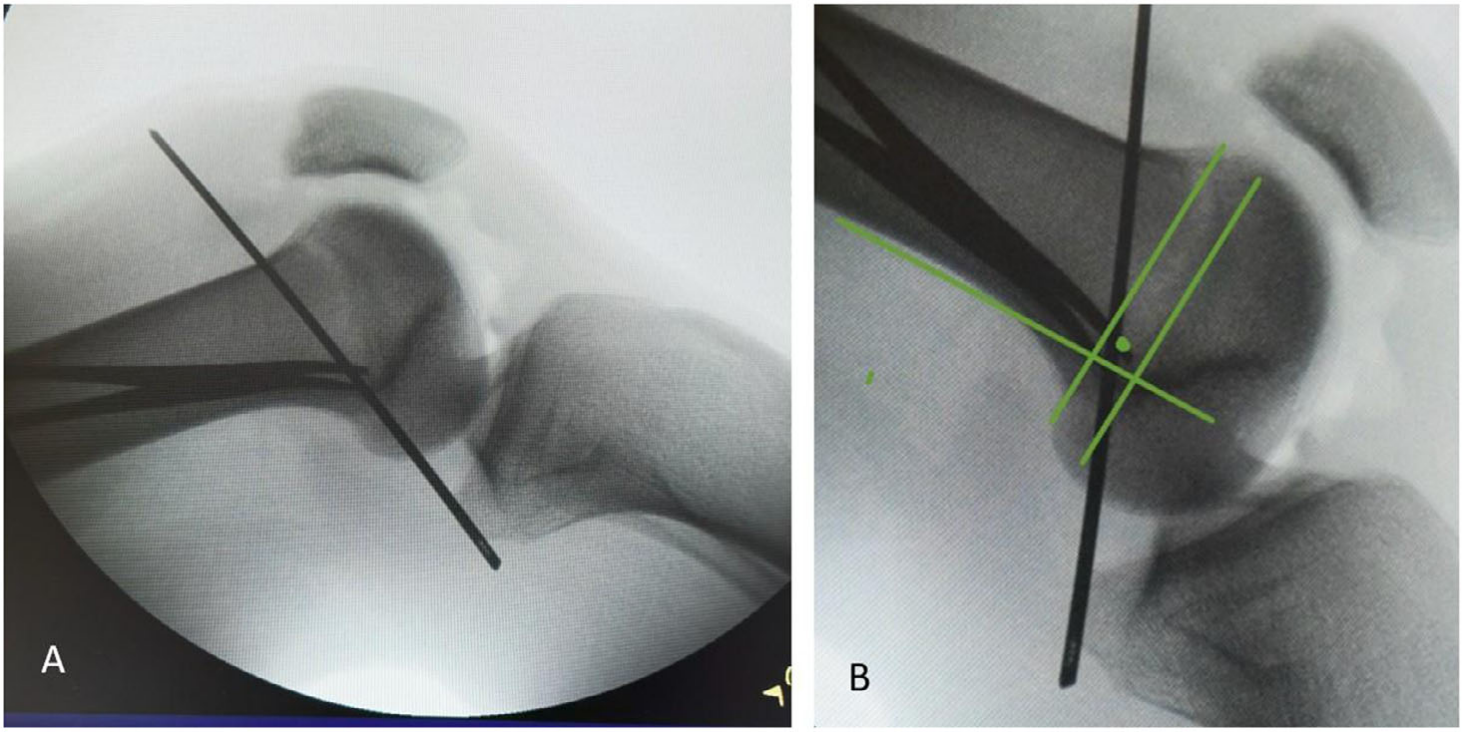
Patient is in the supine position, right knee, and the arthroscopic portal was anteromedial. (A) Lateral fluoroscopic view of the knee obtained, a guide wire is passed through the Schöttle point to create the femoral tunnel of the medial patellofemoral ligament. (B) Lateral fluoroscopic view of the knee with anatomical markings and the guide wire in the ideal position (Schöttle point) of the medial patellofemoral ligament femoral tunnel.

**FIGURE 7:**
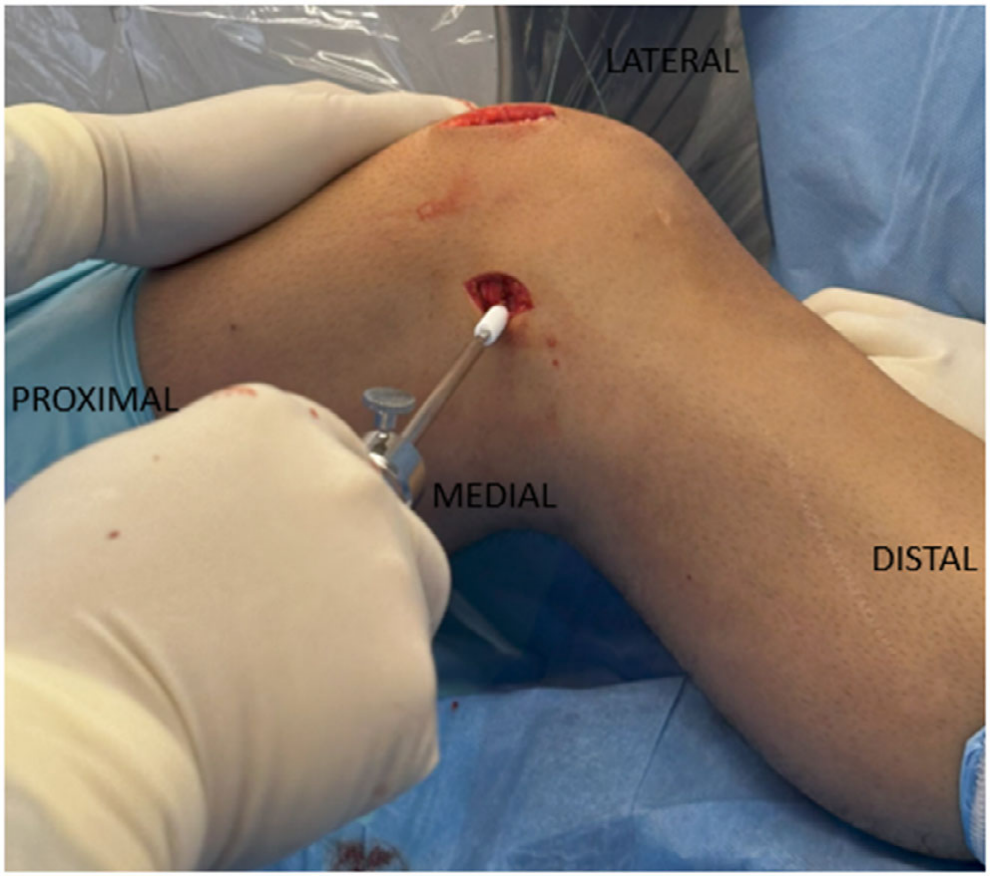
Patiente in supine position, left knee, showing the fixation of the medial patellofemoral ligament graft in the femoral tunnel, with the knee in 30 degrees of flexion using a bioabsorbable screw (Arthrex).

**FIGURE 8:**
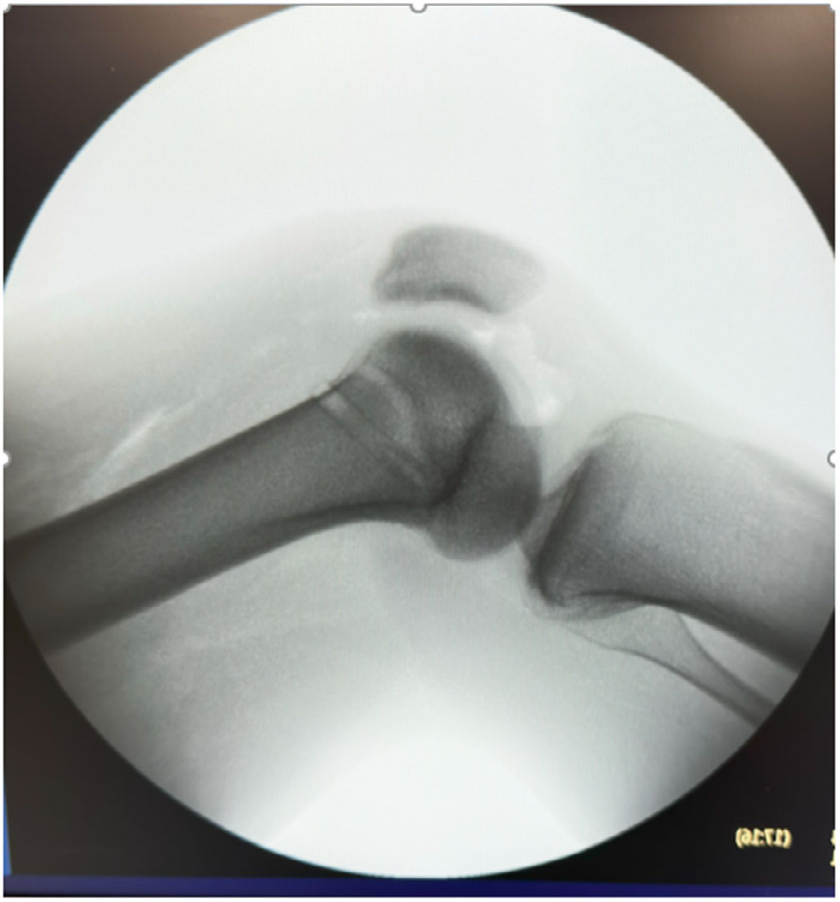
Patient is in the supine position, right knee, and the arthroscopic portal was anteromedial. Final lateral radiograph of the knee showing the reconstruction of the medial patellofemoral ligament with a double graft from the rectus femoris tendon.

The prepared graft is tunneled through the second layer of the knee (between the joint capsule and medial retinaculum) toward the medial epicondyle femur (Figure 5, Video 1). With the knee flexed and a lateral fluoroscopic view obtained, a guidewire is passed through Schöttle's point (Video 1, Figure 6 and Table 2).^23^, ^24^ A femoral tunnel is then drilled at the predetermined anatomical location. With the knee at approximately 30° of flexion, the graft is transferred into the femoral tunnel and fixed with a bioabsorbable screw (Arthrex, Naples, FL, USA) (Video 1, Figures 7 and 8). Adequate tension is applied to avoid laxity or overtightening. Patellar medial stability and full range of motion are confirmed before layered closure and compressive dressing application.

**TABLE 2:** Pearls and Pitfalls of MPFL Reconstruction With RF Graft

**Pearls**	**Pitfalls**
• Perform subperiosteal dissection at the patellar insertion. • Confirm femoral tunnel position using guidewires under fluoroscopy before drilling.	• Avoid MPFL over‐tensioning. • Harvesting a strip of rectus femoris that is too narrow or short. Incorrect femoral tunnel positioning for the MPFL.

MPFL, medial patellofemoral ligament; RF, rectus femoris.

## DISCUSSION

The use of the quadriceps tendon in MPFL reconstruction is based on its structural similarities to the native ligament. Cadaveric studies have shown that a quadriceps tendon strip measuring approximately 10 mm in width and 3 mm in thickness presents biomechanical properties equivalent to the native MPFL in terms of strength and elastic behavior.^10^, ^16^, ^17^, ^18^, ^19^, ^20^ Preliminary results from the present study also support our hypothesis that MPFL reconstruction using the quadriceps tendon may more closely reproduce the structural characteristics of an intact MPFL.^16^, ^17^, ^18^, ^20^

Compared with traditional techniques using hamstring tendon grafts, the present technique avoids the need for patellar tunnels or fixation, reducing the risk of complications such as fractures and fixation failures^4^, ^6^ (Table 1). Furthermore, by maintaining the graft's patellar insertion, the reconstruction tends to be more anatomic and potentially more isometric throughout the range of motion, respecting the original alignment and function of the quadriceps tendon. This minimally invasive approach can be particularly advantageous in skeletally immature patients and in revision surgeries, where preservation of patellar bone integrity is desirable.

## DISCLOSURES

The authors (V.B.M., S.S.B., S.M.G.C., L.C.F., C.P.H., V.B.M.M., P.B.J.) declare that they have no known competing financial interests or personal relationships that could have appeared to influence the work reported in this article.
